# Facile fabrication of self-assembled ZnO nanowire network channels and its gate-controlled UV detection

**DOI:** 10.1186/s11671-018-2774-0

**Published:** 2018-12-24

**Authors:** Hochan Chang, Do Hoon Lee, Hyun Soo Kim, Jonghyurk Park, Byung Yang Lee

**Affiliations:** 1 Department of Mechanical Engineering, Korea University, Seoul, 02841 South Korea; 2 Electronics and Telecommunications Research Institute, Daejeon, 34129 South Korea

**Keywords:** Zinc oxide nanowires, Self-assembly, Heat treatment, Photodetectors

## Abstract

We demonstrate a facile way to fabricate an array of gate-controllable UV sensors based on assembled zinc oxide nanowire (ZnO NW) network field-effect transistor (FET). This was realized by combining both molecular surface programmed patterning and selective NW assembly on the polar regions avoiding the nonpolar regions, followed by heat treatment at 300 °C to ensure stable contact between NWs. The ZnO NW network FET devices showed typical n-type characteristic with an on-off ratio of 10^5^, transconductance around 47 nS, and mobility around 0.175 cm^2^ V^− 1^ s^− 1^. In addition, the devices showed photoresponsive behavior to UV light that can be controlled by the applied gate voltage. The photoresponsivity was found to be linearly proportional to the channel voltage *V*_ds_, showing maximum photoresponsivity at *V*_ds_ = 7 V.

**Electronic supplementary material:**

The online version of this article (10.1186/s11671-018-2774-0) contains supplementary material, which is available to authorized users.

## Background

One-dimensional nanomaterials are well known to have various advantages over other film-type or bulky materials due to their high specific surface area, well-oriented uniform crystal structures, and directed charge transport paths that enable high device performance and easy device fabrication [1, 2]. In particular, due to their unique dimensionality, semiconducting nanowires (NWs) have been utilized in diverse applications such as energy conversion, memory, optical devices, and sensors [3–9]. Among them, zinc oxide (ZnO) NWs have shown excellent semiconductor characteristics with large direct band gap of 3.37 eV and high exaction binding energy of 60 meV at room temperature [10]. Also, ZnO are known to be environmentally friendly, naturally abundant, and low-cost in production [11]. Hence, ZnO have been applied to a wide variety of fields including light-emitting diodes [12, 13], laser diodes [14], solar cells [15–18], photodetectors [19–23], transparent field-effect transistors [24–26], generators [27, 28], and chemical sensors [29, 30]. Meanwhile, UV sensors based on ZnO NWs have been demonstrated [31–33], but the devices were difficult to fabricate. These methods of fabricating ZnO NW network devices generally include electrode-deposited ZnO NW coating films followed by an etching process to define the channel. This method is difficult to control physical dimensions such as the adjustment of the ZnO channel width. To overcome these problems, a method of hydrothermal growth of ZnO NW on pre-patterned layers has been studied, but additional etching and/or hydrothermal growth processes are required. Currently proposed ZnO nanowire patterning methods such as laser-induced selective growth [34, 35] or hydrothermal growth of ZnO NWs with localized heaters [36] involve high-cost and high-temperature processes. There is also a case where a vertically grown ZnO nanowire array network is used to use aligned nanowire array networks with controllability of device properties [37], but this also requires a lot of effort to require vacuum equipment such as CVD and is not suitable for large area and low-cost production. Some low-temperature process with low-cost production based on microcontact printing [38] or inkjet printing was suggested [39], but the control of the NW density and corresponding device properties still remains as a challenge.

In this work, we demonstrate a highly reproducible and facile method to fabricate arrays of gate-controlled UV sensors based on ZnO NW network field-effect transistor (FET) by using self-assembly on molecularly patterned substrates and heat treatment. The ZnO NW network channels have a device yield of 90% with average resistance values of a few hundred kΩ. The post heat treatment is believed to have the effect of removing the residual organic solvents and enhancing the electrical contact between the NWs. The ZnO NW-based FET devices showed n-type properties with an on-off ratio of 10^5^, transconductance around 47 nS, and mobility around 0.175 cm^2^ V^−1^ s^−1^. The physical properties can be controlled by changing the NW assembly conditions like molecular pattern, NW density in solution, pulling speed, and so on. Finally, we successfully realized arrays of ZnO UV sensors with controllable photoresponsivity and response time by the applied gate voltage. The negative gate voltage applied to the n-type FET minimized the initial current due to the depletion of the ZnO NW channel. Indeed, maximum photoresponsivity to UV light was found at gate voltage below − 55 V and the photoresponsivities were found to be proportional to the channel voltage *V*_ds_, showing maximum photoresponsivity at *V*_ds_ = 7 V. In addition, the negative gate voltage facilitated the device recovery after UV light exposure. It should be noted that, although previous reports on ZnO NW network devices have been reported [34, 35], our ZnO NW devices have ZnO NW structures with controllable channel width and thickness without using any chemical or plasma etching process. This mild process combined with heat treatment below the ZnO recrystallization temperature (~ 400 °C) resulted in large-scale facile fabrication of gate-controlled UV sensors with high on-off ratio and photoresponsivity. We expect that our process and device performance will expedite the commercialization process of ZnO NW-based applications.

## Methods

### ZnO NW network FET fabrication

ZnO NWs of length 2~3 μm and diameter 200 nm were purchased from Sigma-Aldrich, Inc., USA. The NWs were dispersed to 1 wt% concentration in dichlorobenzene (DCB) by sonication for 3 s. For preparing the molecularly patterned substrates, photoresist (AZ 5214E) was patterned on SiO2 (300-nm-thick SiO2 on 500-μm-thick p-doped Si wafer) substrate by typical photolithography method. Then, the substrate was dipped into 1:500 *v*/*v* octadecyltrichlorosilane (OTS) in hexane about 3 min [40]. During this process, a monolayer of OTS molecules was self-assembled on the surface of the exposed SiO_2_ region to create a non-polar OTS region. After OTS treatment, the substrate was immersed in acetone for 2 min to remove the region protected by photoresist, exposing the polar SiO_2_ regions on which the ZnO NWs are to be assembled. The self-assembled OTS monolayers have methyl-termination that makes it a non-polar region. On the other hand, the SiO2 surface works as a polar region from its hydroxyl groups (OH). For ZnO NW assembly, the substrate was dipped into the NW solution and pulled at a controlled pulling speed in the range 0.5~10 mm min^−1^. The ZnO NW solution was stirred with a magnetic bar during the pulling process at 100 rpm to prevent NW aggregation and precipitation. As the substrate was pulled, evaporation proceeded fastest near the air–suspension–substrate interface resulting in the selective adsorption of ZnO NWs on the polar SiO_2_ region due to van der Waals force, while avoiding the nonpolar OTS regions. After ZnO NW assembly on the substrate, electrodes (Ti/Al, 10/300 nm) were deposited by thermal deposition, followed by lift-off process.

### Heat treatment process

The heat treatment was performed at 1 Torr pressure in Ar ambient inside a furnace. The temperature was raised to 110 °C during 3 min and kept constant for 10 min in order to evaporate any remaining solvents. Then, the temperature was raised to 300 °C during 3 min and kept constant for 10 min to improve the inter-NW interface and reduce the potential barriers and contact resistance between the NWs [41]. Afterwards, the sample was cooled down to room temperature during 1 h and then taken out from the furnace.

### Measurement of the electrical and photoresponsive properties of ZnO NW network FETs

The electrical properties such as I–V characteristics and gate properties were measured using a probe station equipped with a semiconductor parameter analyzer (4200A-SCS, Keithley, USA). The source-drain voltage was scanned from 0 V to 7 V. The gate voltage was swept from − 60 V to + 60 V. From the gate-dependent I–V characteristics, we calculated the transconductance and mobility values [42, 43]. To avoid any ambient effects on the resistance of the NW channels, the temperature and relative humidity were kept constant at 23 ± 1 °C and 35 ± 1%, respectively, during the measurements. For UV photoresponse measurement, the source-drain voltage *V*_ds_ was kept at 7 V. The UV source was a handheld UV lamp (Spectroline ENF-260C/FE, USA) with an excitation wavelength of 365 nm and power density of 350 μW cm^−2^.

## Results and discussion

**Fig. 1:**
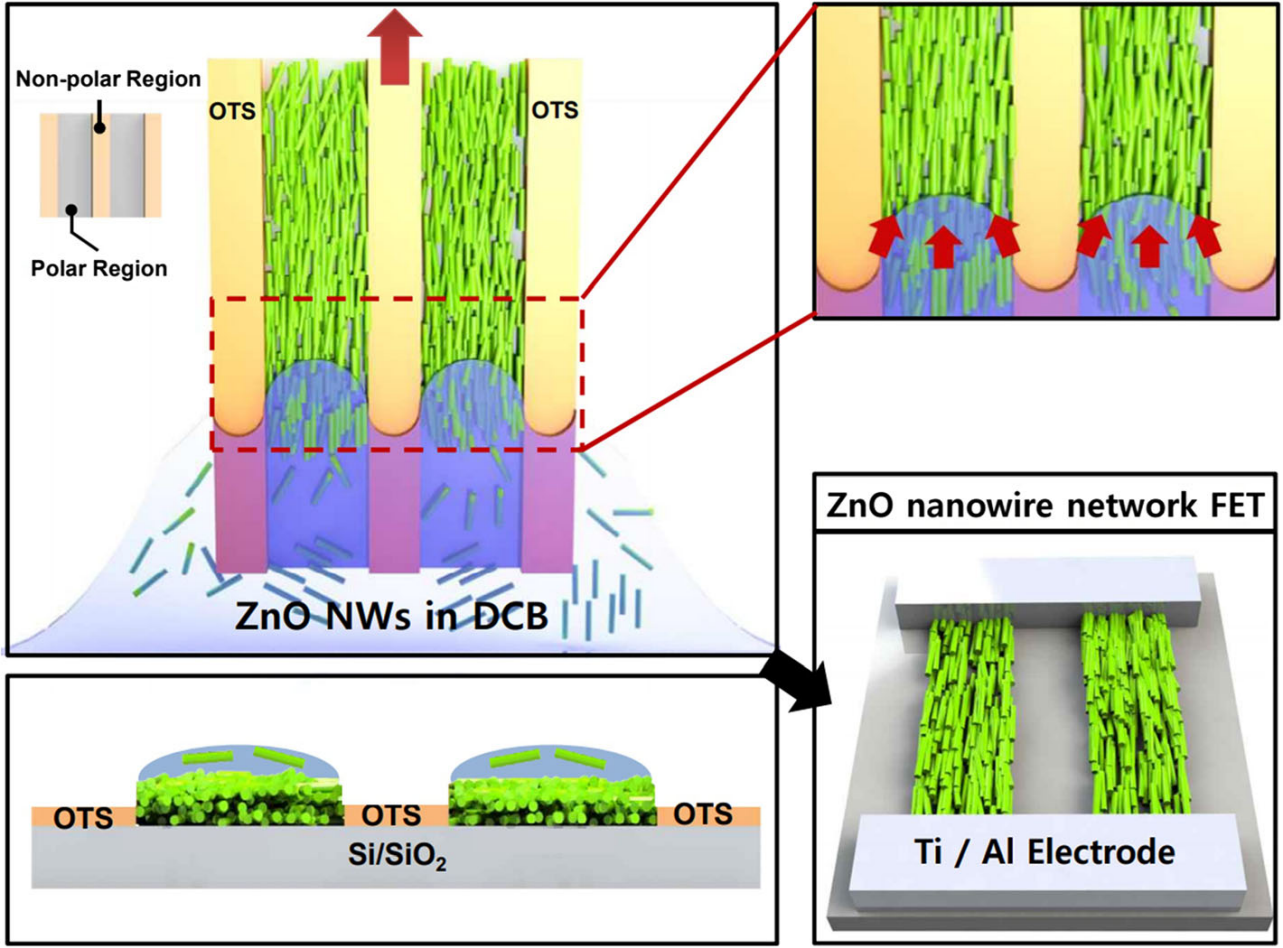
Schematic diagram of the fabrication procedure of ZnO NW network FET. Assembly of ZnO NWs on molecularly patterned substrates using pulling process

**Fig. 2:**
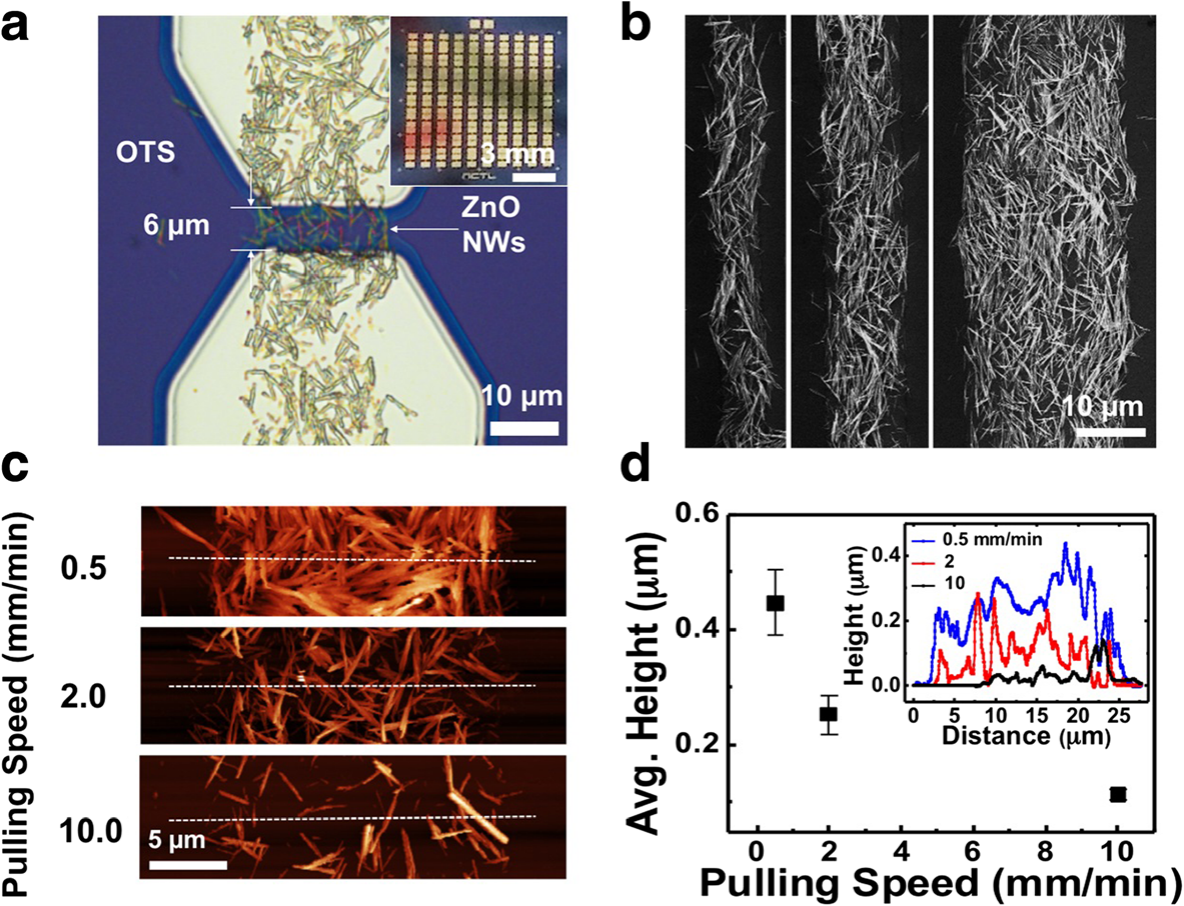
Channel formation and thickness control of ZnO NW network FET devices. **a** Magnified optical image of a 6 μm length NW network channel and Al electrodes. The inset is optical micrograph of 10 × 10 array devices with micro-patterned channels. **b** Scanning electron microscope (SEM) images showing the selective assembly of ZnO NWs to line patterns with diverse line widths of 5, 10, and 20 μm. **c** AFM image of ZnO NWs network. **d** Average distribution of height vs pulling speed at different pulling speed of 0.5~ 10 mm min^−1^. The inset shows the AFM height profile for different pulling speeds of 0.5, 2, 10 mm min^−1^

Figure 1 shows the schematic diagram describing the preparation of percolating ZnO NW network channels and subsequent heat treatment. First, an OTS-patterned substrate was dipped into ZnO NW suspension (1 wt% in dichlorobenzene) and pulled from ZnO NW suspension using a home-made pulling system at different pulling speeds of 0.5 mm min^−1^ to 10 mm min^−1^ (Additional file 1: Figure S1). During the pulling process, a liquid meniscus containing ZnO NWs was dragged against the OTS-patterned substrate. The ZnO NWs assembled exclusively on the exposed SiO_2_ channel regions. As shown in the inset of Fig. 2a, a total of 100 devices were fabricated on 4-in.-wafers using our fabrication method. Figure 2a shows the optical image of a percolating ZnO NW network channel, and the inset shows the FET device array. The average diameter of a ZnO NW was 200 nm, NW length was 2~3 μm, the channel length and width were 6 μm and 20 μm, respectively. After ZnO NW assembly, the source-drain electrodes were made by a conventional photolithography technique, thermal deposition of metal (10 nm Ti, 300 nm Al) and lift-off process.

As shown in Fig. 2b, we were able to obtain ZnO NW patterns with diverse line widths of 5, 10, and 20 μm by changing the SiO_2_ region pattern size. The surrounding OTS regions are non-polar due to the methyl terminals of the OTS molecules. The NWs are thought to get adsorbed only on the polar SiO_2_ regions by van der Waals interaction [40]. The selective assembly of ZnO NWs was also confirmed with energy-dispersive X-ray spectroscopy (EDS) (see Additional file 1: Figure S2). Here, the Zn signals were confined to those regions with ZnO NWs.

The physical properties of percolating ZnO NW network channels such as thickness and density were controlled by modulating the substrate pulling speed from the NW solution during NW assembly. Figure 2c shows the AFM (atomic force microscopy) images of ZnO NW networks assembled at different pulling speeds of 0.5, 2, and 10 mm min^−1^. The average height profile vs pulling speed is shown in Fig. 2d. The NW density was 1.21 NW μm^−2^ at pulling speed 0.5 mm min^−1^, and 0.09 NW μm^−2^ at 10 mm min^−1^. The NW channel thickness increased by reducing the pulling speed. The height of the NW channel was usually about 1.5~2 times higher than single NW average diameter of 200 nm at the slowest speed rate 0.5 mm min^−1^ (Fig. 2d, inset). At pulling speed of 10 mm min^−1^, the network connection reached the percolation limit, beyond which the network showed no connection. Current methods of fabricating ZnO NW network devices generally involve electrode deposition ZnO NW-coated film, followed with some kind of etching process to define the channels [38, 39]. This method is difficult to control the physical dimensions such as adjustment of the ZnO channel width. To overcome these problems, a method using hydrothermal growth of ZnO NWs on pre-patterned layers has been studied [44, 45], but it requires also additional etching process and/or hydrothermal growing processes that take time and cost. In contrast, our method can easily control the width and length of a channel by previously patterning the channel with OTS molecules and then assembling the NWs through a pulling system.

**Fig. 3:**
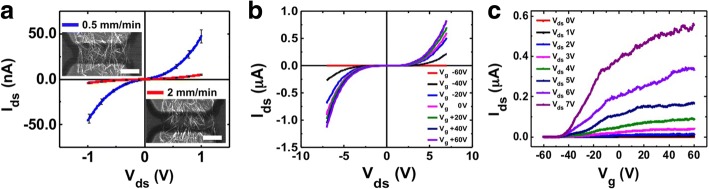
Connectivity and electrical properties of ZnO NW network by control of pulling speed. **a** ZnO NWs network FET electrical properties of pulling speed 0.5 and 2 mm min^−1^. Typical current-voltage characteristics of devices fabricated at different pulling speeds of 0.5 and 2 mm min^−1^. The network channels showed Schottky contact from − 1 to 1 V. The insets are SEM images of network channel at 0.5 (top left) and 2 (bottom right) mm min^−1^. The scale bars are 10 μm for both cases. **b** Current-voltage characteristics of various back-gate voltage. *V*_g_ ranged from − 60 V to 60 V in 20 V steps. **c** *I*_ds_ vs *V*_g_ relations of ZnO NWs network channel fabricated at various *V*_ds_. *V*_ds_ ranged from 0 to 7 V in 1 V steps

The electrical properties can be also controlled by modulating the pulling speed. Figure 3 shows the electrical properties before heat treatment. Figure 3a shows the change of I–V characteristics with different pulling speeds. When the pulling speed was decreased from 2 to 0.5 mm min^−1^, the initial current increased from 5 to 50 nA at 1 V. This is presumably due to the increased network connectivity with increased NW density in the channel. The typical gate-dependent I–V characteristic curves of a ZnO NW FET fabricated at 2 mm min^−1^ pulling speed are shown in Fig. 3b, c. Figure 3b displays the I–V characteristics at different gate voltage *V*_g_ values (from − 60 V to 60 V in 20 V steps). The *I*_ds_-*V*_*g*_ gate characteristics in Fig. 3c show typical n-type characteristics with an increased on-off ratio by five orders of magnitude from the off current of 3 pA to 556 nA and decreased off-current when the pulling speed was increased from 0.5 to 2 mm min^−1^ (see Additional file 1: Figure S3). This increase of on-off ratio with decreased film density can be explained by noting that the channel is more affected by the electrical field from the back gate as we make the NW channel thinner [46]. Also, the pulling speed has an effect on the device yield and two-probe resistance frequency distribution (Additional file 1: Figure S4). The resistance shows the average value of 28.2 ± 4 MΩ and ~ 92% yield at 0.5 mm min^−1^. However, the distribution shifts to 877 ± 280 MΩ and ~ 78% yield at 2 mm min^−1^ pulling speed. Here, the yield is defined as the number fraction of devices with measurable resistance values above equipment noise.

Here, the gate characteristic of the FET does not have a clean saturation regime. According to previous reports, the ZnO NW network did not exhibit clean saturation regime, possibly due to the increased carrier scattering by complex NW network path, large surface area, and grain boundaries at NW junctions [47]. Our ZnO NW network forms a number of path between a source and drain. Also, ZnO NW network channel having a thickness up to about 0.4 μm (Fig. 2d). The non-uniform thickness of nanowire cause different distance to the gate for each nanowire, and the degree of modulation is slightly different. Therefore, the FET characteristic does not have a clean saturation regime like a single nanowire FET.

**Fig. 4:**
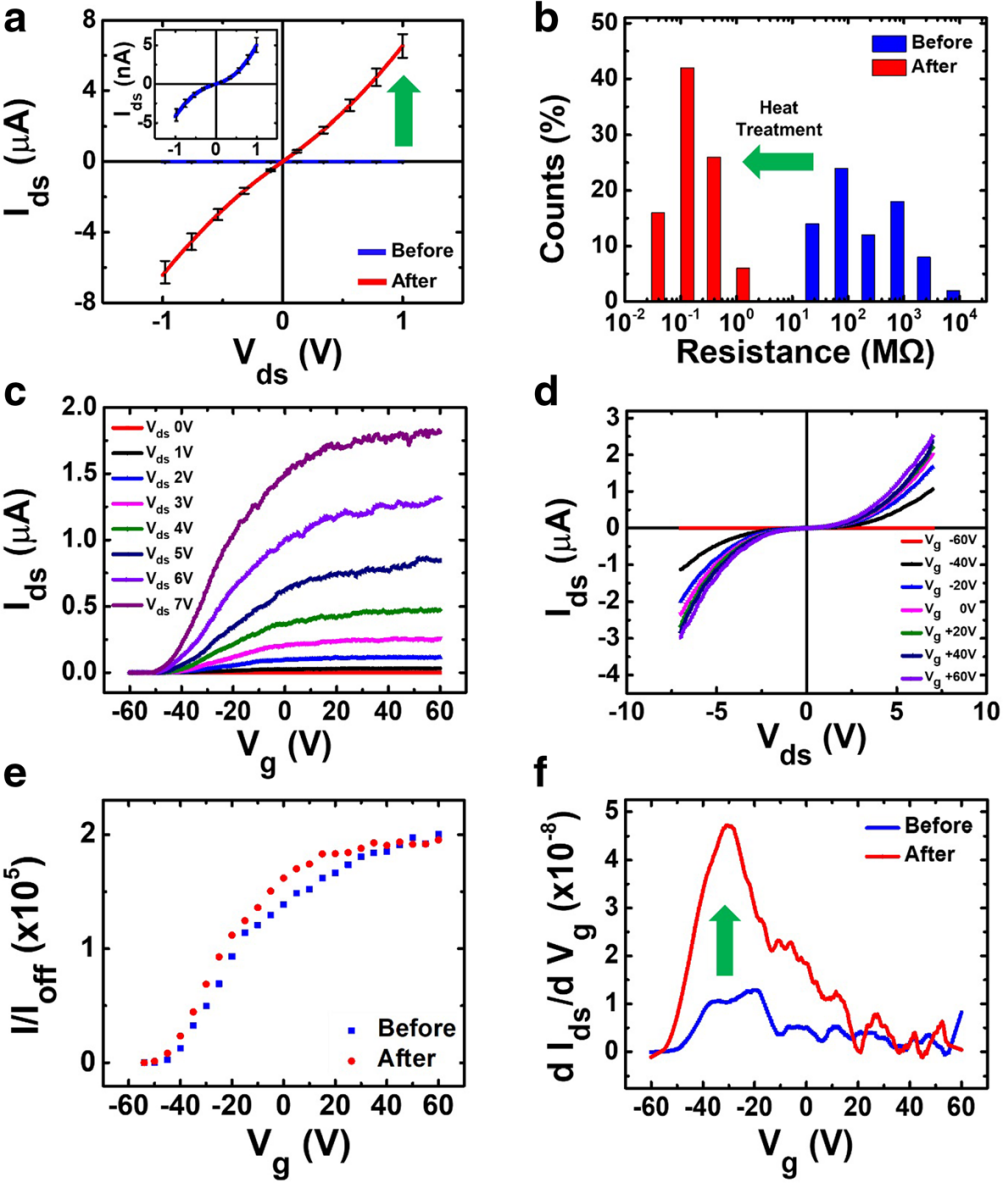
ZnO NWs network electrical properties after heat treatment. **a** Typical current-voltage characteristics before and after annealing of pulling speed 2 mm min^−1^. The network channels showed Schottky contact behavior. (inset) I–V characteristics before annealing, magnified. **b** Resistance frequency distribution of ZnO NWs network pulled at speed of 2 mm min^−1^. Average resistance decreased for about three orders. (**c**) I–V characteristics at different back-gate voltages. *V*_g_ ranged from − 60 V to 60 V in 20 V steps. **d** Electrical properties of *I*_ds_ vs *V*_g_ after heat treatment. **e** Comparison of *I*/*I*_off_ ratio at different *V*_g_ values, before and after heat treatment (*V*_g_ step = 5 V). **f** Improved transconductance by heat treatment

The electrical properties of as-produced devices can be enhanced by subsequent heat treatment process to improve the uniformity in electrical properties and further lower the contact resistance between the NWs [41]. The heat treatment was performed in low-pressure conditions at 300 °C for 10 min while flowing Ar gas at 100 sccm (see Additional file 1: Figure S5). Figure 4 shows the electrical property change of the samples fabricated at 2 mm min^−1^ pulling speed. After the heat treatment, the current at 1 V bias increased from 600 nA to 6.5 μA (Fig. 4a). The resistance frequency distribution in Fig. 4b shows a drop of the average resistance from 877 ± 280 MΩ to 207 ± 37 kΩ, about 3 order of magnitude. Also, the device yield increased from 78 to 90%, presumably due to the enhanced electrical contact between NWs. We focused on using the advantages of NW connection enhancement through heat treatment. For this reason, the temperature was not raised to more than 400 °C where ZnO recrystallizes. Such recrystallization has been reported to affect the oxygen desorption and adsorption characteristics at the ZnO surface during UV illumination [41]. Therefore, in order to obtain only the improvement of the connection between the NWs through the heat treatment, heat treatment was performed up to 300 °C to improve the interface between the NWs. This resulted in enhancing electrical stability and characteristics. We believe that our heat treatment process might remove the adsorbing molecules such as moisture or hexamethylenetetramine (HMTA), since our process temperature is higher than the melting point of HMTA (290 °C). This resulted in enhancing the NW FET performance because it improves the junction between the NWs and removes other adsorbing molecules that degrade the performance of the NWs such as moisture.

The typical *I*_ds_-*V*_ds_ and *I*_ds_-*V*_*g*_ characteristic curves of a ZnO NW FET are shown in Fig. 4c, d. Figure 4e shows that *I*_ds_-*V*_*g*_ characteristic curves are similar before and after heat treatment, and the maximum *I*_on_*/I*_off_ ratio is ~ 2 × 10^5^. This indicates that the heat treatment only improves the connection between the NWs to lower the resistance and does not cause a change in intrinsic electrical properties. Figure 4f shows the improvement of the transconductance *dI*_ds_*/dV*_*g*_ after heat treatment, which can be attributed to the enhanced electron mobility in the ZnO NW device. The maximum transconductance (*g*_*m*_ *= dI*_ds_*/dV*_*g*_) was extracted from the maximum slope of the *I*_ds_-*V*_*g*_ characteristics and the maximum on-off ratio at 7 V of V_ds_. (Additional file 1: Figure S6). The calculated maximum transconductance was ~ 47 nS at *V*_*g*_ = − 30 V. We used the formula *μ* = *g*_*m*_·*L*/(*W*·*C*_*d*_·*V*_ds_) for the estimated mobility calculation [48]. The mobility was calculated to be 0.175 cm^2^ V^−1^ s^−1^. This is comparable to previously reported values of 0.018 cm^2^ V^−1^ s^−1^ using ZnO NWs device array [49].

**Fig. 5:**
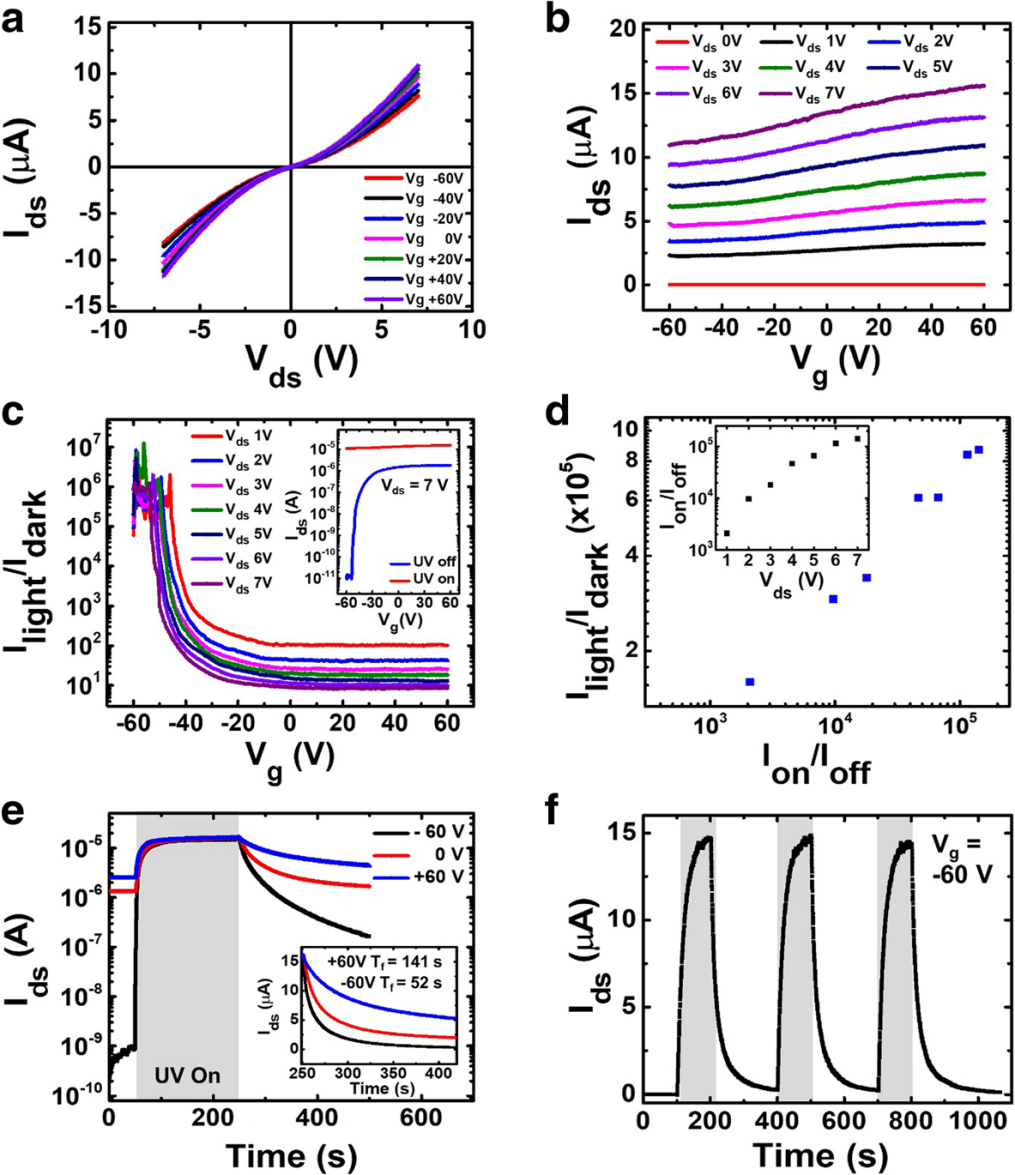
UV sensing characteristics of ZnO NW FETs. **a** I–V characteristics under UV illumination at different back-gate voltages. *V*_g_ ranged from − 60 V to 60 V in 20 V steps. **b** Electrical properties of I_ds_ vs V_g_ under UV illumination. **c** *I*_lignt_/*I*_dark_ as a change of gate voltage. Maximum *I*_lignt_/*I*_dark_ was obtained around *V*g ~ − 55 V. Inset, *I*_ds_-*V*_g_ characteristics under UV illumination and under darkness. **d** Linear relationship between photo/dark current ratio (*I*_lignt_/*I*_dark_) and initial on-off current ratio (*I*_on_/*I*_off_) of the ZnO NW FET. The inset shows the initial *I*_on_/*I*_off_ ratio for various *V*_ds_. The maximum *I*_on_/*I*_off_ ratio was at *V*_ds_ = 7 V. **e** Photoresponse of ZnO NW network FET photodetector with and without UV illumination in the air. The inset shows exponential decay characteristics after the UV light off. **f** Time-resolved photoresponse of the ZnO NW network channel devices recorded by switching on and off the UV light

Finally, we observed the UV photoresponse of the ZnO network FETs and its dependence on the gate voltage. Figure 5a shows the I–V characteristics with UV illumination at a different gate voltage (from − 60 V to 60 V, in 20 V steps). The *I*_ds_*-V*_*g*_ characteristics under UV illumination in Fig. 5b shows a decreased on-off ratio. The UV light had the effect of increasing the off-current of the n-type FET device by creating a photoexcited carrier. Figure 5c shows the difference of measured current for the UV light on and off condition. The UV photoresponsivity (*I*_light_*/I*_dark_: ratio of photocurrent to dark current) varies depending on the applied gate voltage and shows the maximum ratio value of 8.6 × 10^5^ at V_g_ − 55 V or less. The inset of Fig. 5c shows *I*_ds_-*V*_g_ characteristics with and without UV illumination when V_ds_ = 7 V (*V*_ds_*-I*_ds_ characteristics show in Additional file 1: Figure S7). Figure 5d shows a linear relationship between the *I*_light_*/I*_dark_ and the on-off current ratio (*I*_on_*/I*_off_). The *I*_on_*/I*_off_ increase leads to the improvement of the UV photoresponsivity. To show the improvement with an increase of the current value *V*_*ds*_, we plot the data of Fig. 5d as an on-off value according to the current (inset). Then, the *V*_*g*_ = − 60 V and *V*_*ds*_ = 7 V condition was the optimal condition where the *I*_light_*/I*_dark_ ratio was maximum when comparing before and after UV illumination.

*I*_ds_*-V*_*g*_ characteristics under UV light showed that the transistor changed from a semiconducting state (Fig. 4c) to a conducting (accumulation) state (Fig. 5b). This change can be expected to increase the photoexcited carrier concentration to a degenerate level under UV light [50]. The *I*_light_/*I*_dark_ ratios of our devices were about 2 × 10^4^, 10 and 6, at gate voltages of − 60 V, 0 V, and 60 V, respectively (Fig. 5e). This shows that the UV photoresponsivity can be adjusted by the gate voltage. As *V*_g_ decreased, the photoresponsivity increased.

We compared the photoresponsivity performance of ZnO NW network-based photodetectors from other studies. For example, CVD grown ZnO nanowire arrays showed UV photoresponsivity (*I*_light_/*I*_dark_) of ~ 10^4^ [33, 51]. In our case, we could achieve a similar photodetector responsivity of 2 × 10^4^ without using any high temperature and/or high vacuum processes. Other studies using methods such as inkjet printing [47] or vertically aligned nanowires [52] showed photoresponsivity levels of 10^3^ to 10^4^, which are the comparable or slightly lower than our study (see Additional file 1: Figure S8). Furthermore, our research shows gate-controllable characteristics, which is advantageous in tuning the device sensitivity according to light conditions.

The UV response of ZnO NWs can be explained by depletion region modulation resulting from oxygen desorption and adsorption [53]. UV light causes desorption of the oxygen ions adsorbed on the ZnO NW surface. The oxygen desorption increases the effective channel thickness, resulting in increased current through the NWs. In addition, reduction of the desorption region due to oxygen desorption by UV light lowers the junction barrier height between the NWs, which makes the current flow drift more efficient [54, 55]. Because our devices exhibited n-type semiconducting behaviors, the dark current was minimized at large negative *V*_g_. Therefore, the photoresponsivity at the large negative gate voltage was maximized (see Additional file 1: Figure S9).

In addition, the gate voltage affects the recovery time to the initial state when the UV light is turned off. The fall (decay) time when *V*_g_ = − 60 V and + 60 V are 52 s and 141 s, respectively, showing the difference by three times (inset, Fig. 5e). The time at which the current increases (rise time) or decreases (fall time) from 10% to 90% is defined as the recovery time. The electric field due to the gate bias affects the recombination possibility of electrons and holes in the absorption process of oxygen molecules which was desorbed by UV light [56, 57]. This is involved in the time to return to the initial state of the device. Therefore, the recovery time could be delayed or short depending on the electric field. Figure 5f shows the repetitive photoresponse by applying *V*_g_ = − 60 V. This shows the time-resolved photoresponse of the ZnO NW network channel devices recorded by switching on and off the UV light. We confirmed that no degradation of photoresponsivity occurs for repetitive UV responses.

## Conclusions

We demonstrated an effective fabrication method of arrays of gate-controlled UV sensors using ZnO NW FETs. Our ZnO NW devices have ZnO NW structures with controllable channel width and thickness without using any chemical or plasma etching process, this mild process combined with heat treatment below the ZnO recrystallization temperature (~ 400 °C) resulted in large-scale facile fabrication of gate-controlled UV sensors with high on-off ratio and photoresponsivity with a device yield of 90%. The fabricated ZnO NWs network UV sensors show n-type gate properties with on-off ratio 10^5^, transconductance around 47 nS, and mobility around 0.175 cm^2^ V^−1^ s^−1^. These electrical properties can be modulated by process parameters in the pulling method such as pulling speed. The electrical properties can be further enhanced with heat treatment method. The devices show high sensitivity to UV light, and the photoresponsivity and response time can be controlled by gate voltage. We expect that our process and device performance will expedite the commercialization process of ZnO NW-based applications.

## Additional file

Additional file 1: **Figure S1.** Home-made pulling system consisted of syringe pump, stirrer. The substrate was pulled vertically. **Figure S2.** Energy dispersive spectroscopy (EDS) mapping image of ZnO NWs network. (a) SEM image of ZnO NWs network. (b) EDS mapping of Zn. (c) EDS data of ZnO NW network channel fixed on SiO_2_ wafer. The peaks show the Zn and O element, respectively. The Si peak is due to SiO_2_ wafer. **Figure S3.** ZnO NWs network electrical properties by controlled pulling speed 0.5 mm min^− 1^. (a) current-voltage characteristics of various back-gate voltage. V_g_ ranged from − 60 V to 60 V in 20 V steps. (b) I_ds_ vs V_g_ relations of ZnO NWs network channel fabricated at various V_ds_. V_ds_ ranged from 0 to 7 V in 1 V steps. **Figure S4.** Resistance distribution of ZnO NWs network devices at different pulling speeds. **Figure S5.** Thermal treatment process of ZnO NW network FET in vacuum condition. The thermal treatment process gives the flow of the Ar gas of 100 sccm rate. Two step raises of the temperature 110 °C to 300 °C. **Figure S6.** Transconductance vs V_g_. The maximum transconductance g_m_ value is 47 nS at Vds 7 V. **Figure S7.** I-V characteristics before (blue) and after (red) UV illumination (Vg = − 60 V). The signal increased by ~ 10^4^ orders. Inset shows a log scale. **Figure S8.** Comparison of performances of ZnO NW network based UV sensors. **Figure S9.** Schematic diagram depicting the carrier generation and transportation processes in the ZnO NW network channel before (left) and after (right) UV illumination. Band diagram of the devices under different gate bias conditions and UV illumination. (DOC 1567 kb)

## Abbreviations

AFM: Atomic force microscopy
DCB: Dichlorobenzene
EDS: Energy-dispersive X-ray spectroscopy
FET: Field-effect transistor
HMTA: Hexamethylenetetramine
NW: Nanowire
OTS: Octadecyltrichlorosilane
SEM: Scanning electron microscope
ZnO: Zinc oxide
