# Supplementary material for: Facile fabrication of self-assembled ZnO nanowire network channels and its gate-controlled UV detection
Source: Nanoscale Res Lett. 2018 Dec 24;13:413. doi: 10.1186/s11671-018-2774-0 (PMC6305260; doi:10.1186/s11671-018-2774-0)
Supplement: Supplementary file 1 — Figure S1. Home-made pulling system consisted of syringe pump, stirrer. The substrate was pulled vertically. Figure S2. Energy dispersive spectroscopy (EDS) mapping image of ZnO NWs network. (a) SEM image of ZnO NWs network. (b) EDS mapping of Zn. (c) EDS data of ZnO NW network channel fixed on SiO2 wafer. The peaks show the Zn and O element, respectively. The Si peak is due to SiO2 wafer. Figure S3. ZnO NWs network electrical properties by controlled pulling speed 0.5 mm min− 1. (a) current-voltage characteristics of various back-gate voltage. Vg ranged from − 60 V to 60 V in 20 V steps. (b) Ids vs Vg relations of ZnO NWs network channel fabricated at various Vds. Vds ranged from 0 to 7 V in 1 V steps. Figure S4. Resistance distribution of ZnO NWs network devices at different pulling speeds. Figure S5. Thermal treatment process of ZnO NW network FET in vacuum condition. The thermal treatment process gives the flow of the Ar gas of 100 sccm rate. Two step raises of the temperature 110 °C to 300 °C. Figure S6. Transconductance vs Vg. The maximum transconductance gm value is 47 nS at Vds 7 V. Figure S7. I-V characteristics before (blue) and after (red) UV illumination (Vg = − 60 V). The signal increased by ~ 104 orders. Inset shows a log scale. Figure S8. Comparison of performances of ZnO NW network based UV sensors. Figure S9. Schematic diagram depicting the carrier generation and transportation processes in the ZnO NW network channel before (left) and after (right) UV illumination. Band diagram of the devices under different gate bias conditions and UV illumination. (DOC 1567 kb) [file 11671_2018_2774_MOESM1_ESM.doc]

Facile Fabrication of Self-assembled ZnO Nanowire Network Channels and its Gate-controlled UV Detection

Hochan Chang^1^, Do Hoon Lee^1^, Hyun Soo Kim^1^, Jonghyurk Park^2*^, Byung Yang Lee^1*^

*Hochan Chang^1^*: hochan@mail.com

*Do Hoon Lee^1^*: dodo0022@naver.com

*Hyun Soo Kim^1^*: kenchan888@korea.ac.kr

*Jonghyurk Park^2*^*: eureka99@etri.re.kr

*Byung Yang Lee^1*^*: blee@korea.ac.kr

^1^ Department of Mechanical Engineering, Korea University, Seoul 02841, Korea

^2^ Electronics and Telecommunications Research Institute, Daejeon 34129, Korea

^*^Corresponding Author: eureka99@etri.re.kr, blee@korea.ac.kr

KEYWORDS: Zinc Oxide Nanowires, Self-Assembly, Heat Treatment, Photodetectors.

SUPPLEMENTARY FIGURES


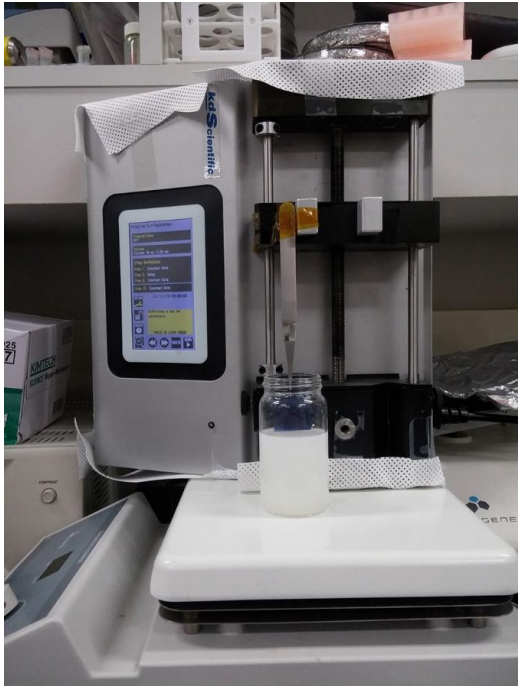


**Figure S1.** Home-made pulling system consisted of syringe pump, stirrer. The substrate was pulled vertically.


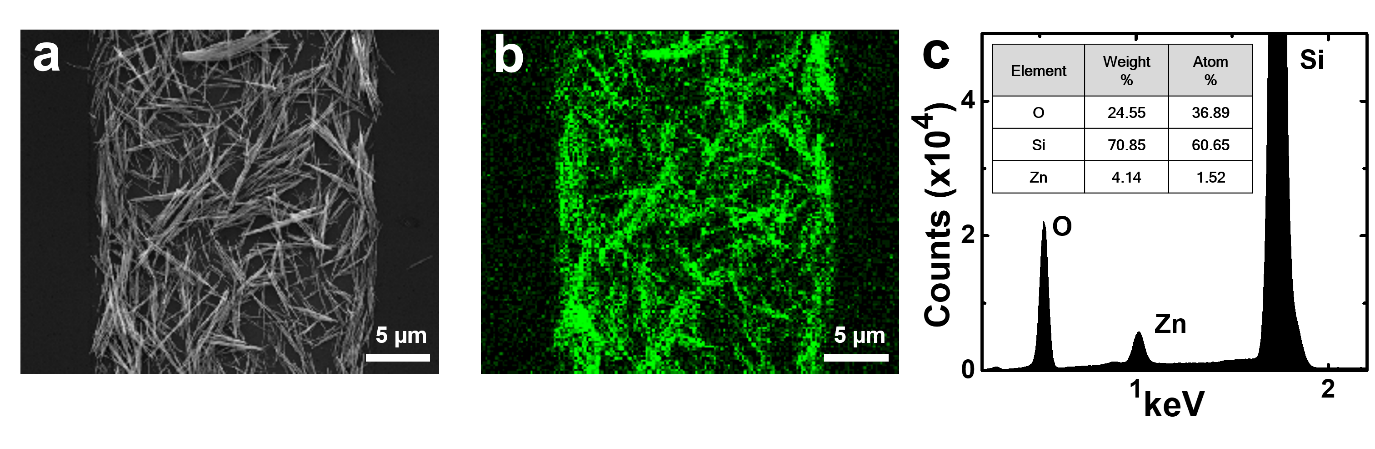


**Figure S2.** Energy dispersive spectroscopy (EDS) mapping image of ZnO NWs network. (a) SEM image of ZnO NWs network. (b) EDS mapping of Zn. (c) EDS data of ZnO NW network channel fixed on SiO_2_ wafer. The peaks show the Zn and O element, respectively. The Si peak is due to SiO_2_ wafer.


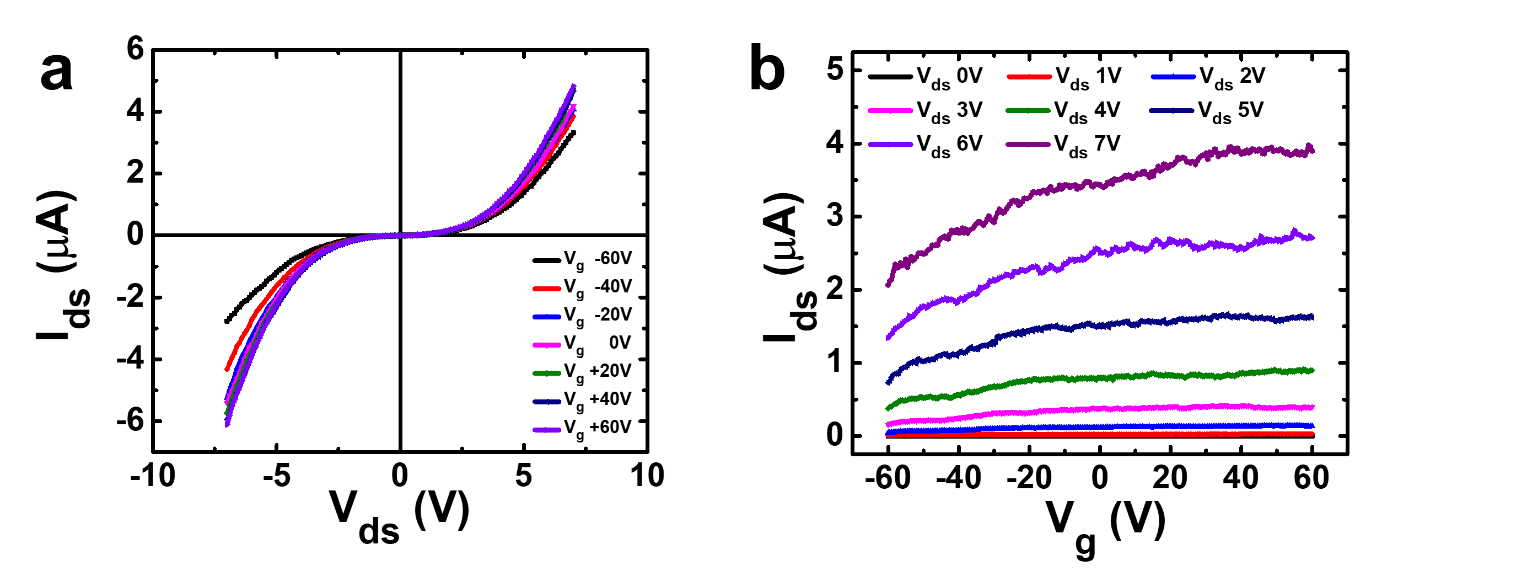


**Figure S3.** ZnO NWs network electrical properties by controlled pulling speed 0.5 mm min^-1^. (a) current-voltage characteristics of various back-gate voltage. V_g_ ranged from -60 V to 60 V in 20 V steps. (b) I_ds_ vs V_g_ relations of ZnO NWs network channel fabricated at various V_ds_. V_ds_ ranged from 0 to 7 V in 1 V steps.


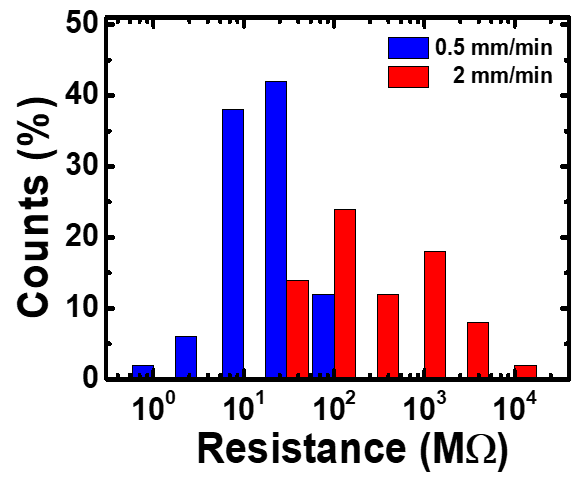


**Figure S4.** Resistance distribution of ZnO NWs network devices at different pulling speeds.


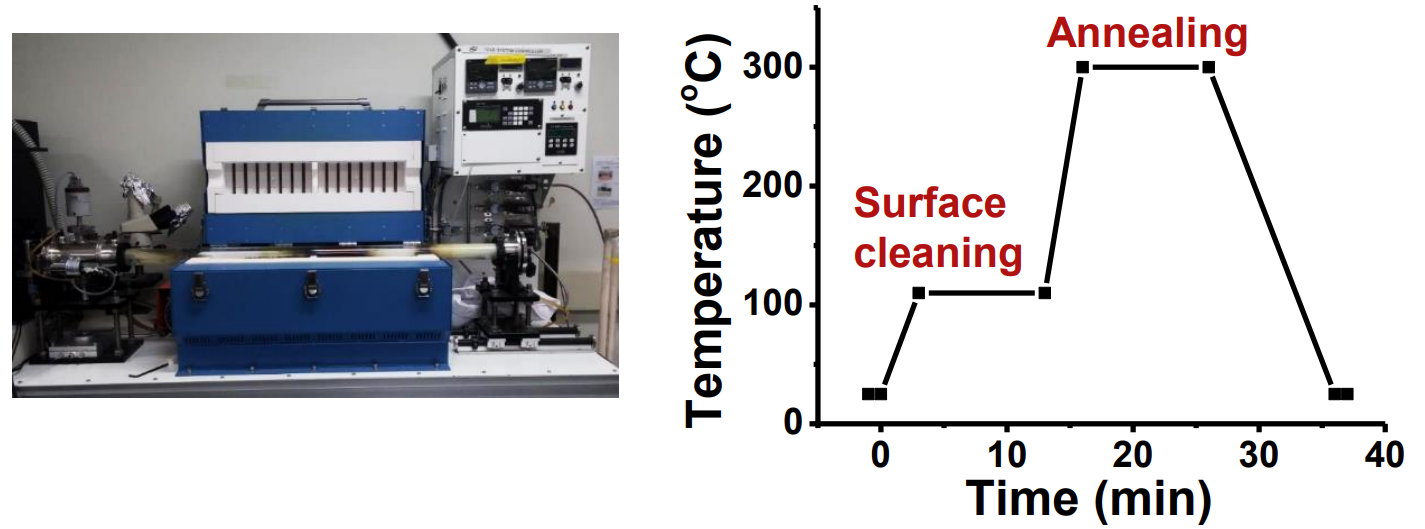


**Figure S5.** Thermal treatment process of ZnO NW network FET in vacuum condition. The thermal treatment process gives the flow of the Ar gas of 100 sccm rate. Two step raises of the temperature 110 °C to 300 °C.


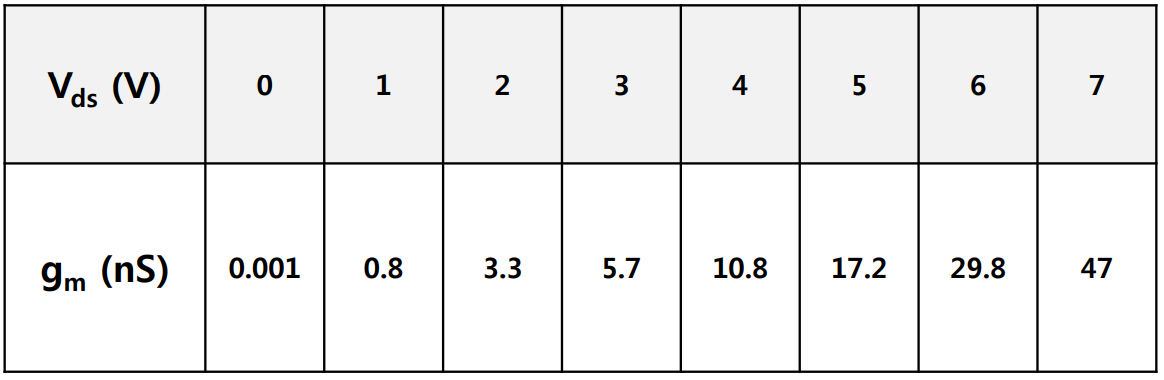


**Figure S6.** Transconductance vs V_g_. The maximum transconductance g_m_ value is 47 nS at Vds 7 V.


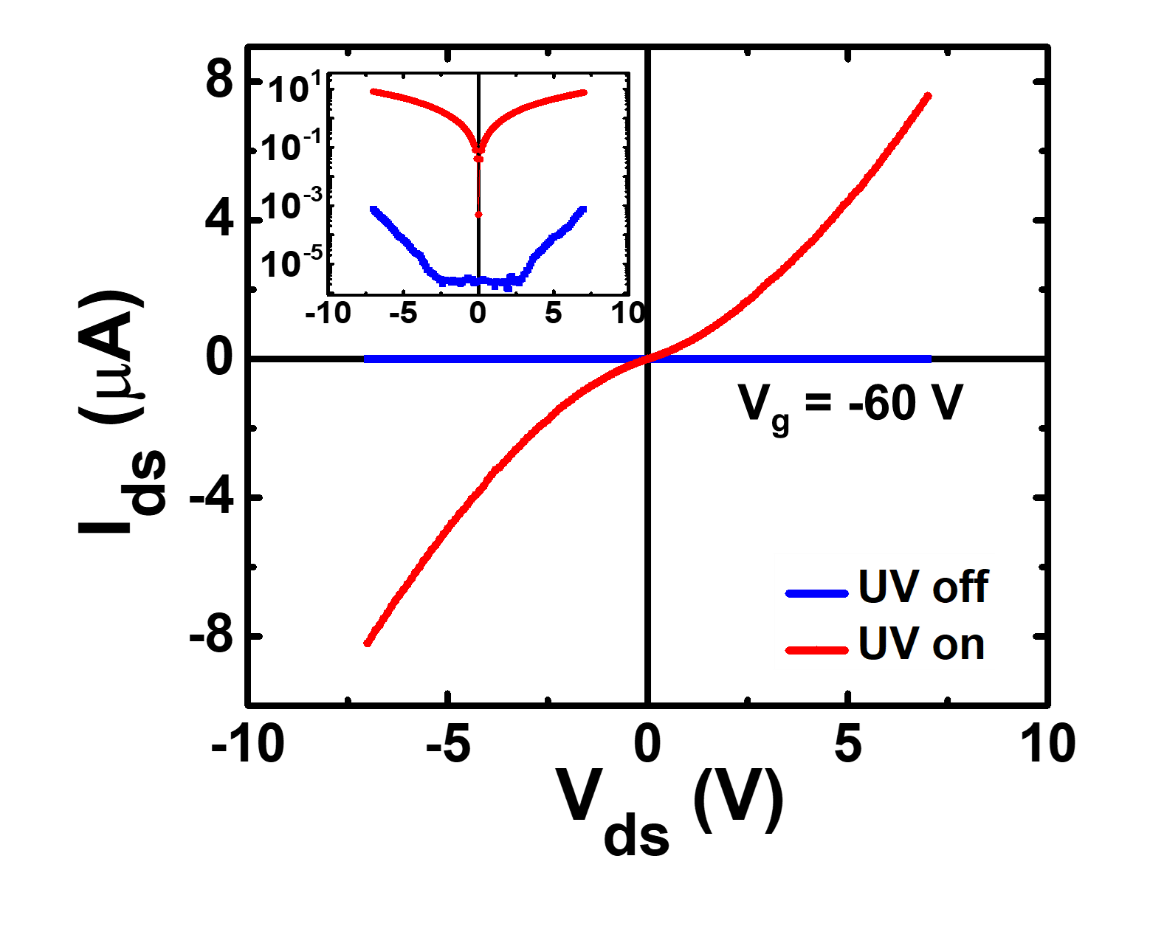


**Figure S7.** I-V characteristics before (blue) and after (red) UV illumination (Vg = -60 V). The signal increased by ~10^4^ orders. Inset shows a log scale.


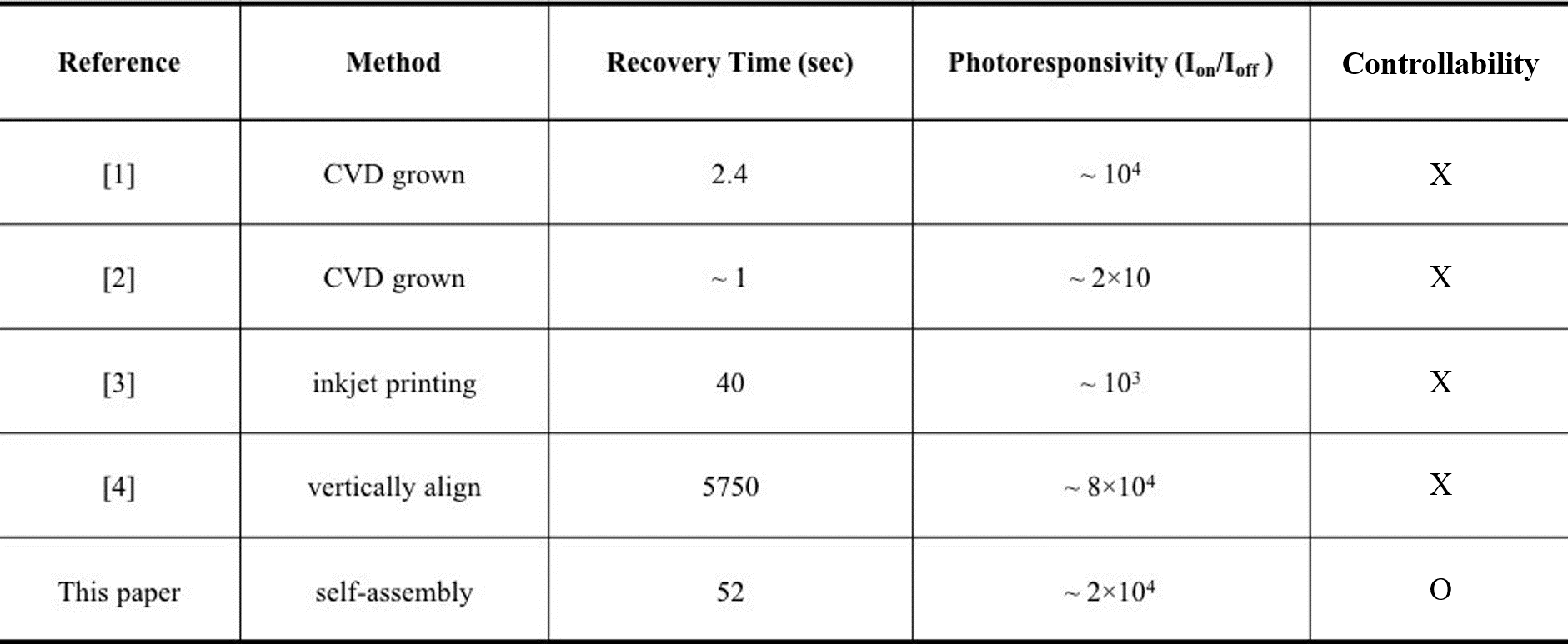


**Figure S8.** Comparison of performances of ZnO NW network based UV sensors.

**References**

[1] Y. Li, A. Paulsen, I. Yamada, Y. Koide, J.-J. Delaunay (2010). Bascule nanobridges self-assembled with ZnO nanowires as double Schottky barrier UV switches. Nanotechnology, 21:295502

[2] P. He, S. Feng, S. Liu, Q. Li, J. Qi, Z. Zhan, X. Lia, Z. Lia, J. Shen, W. Lu (2015). Ultrafast UV response detectors based on multi-channel ZnO nanowire networks. RSC Adv., 5:105288-105291

[3] J. Kwon, S. Hong, H. Lee, J. Yeo, S. Lee, S.-H. Ko (2013) Direct selective growth of ZnO nanowire arrays from inkjet-printed zinc acetate precursor on a heated substrate. Nanoscale Res. Lett., 8:489.

[4] S. Bai, W. Wu, Y. Qin, N. Cui, D. J. Bayerl, X. Wang (2011) High‐performance integrated ZnO nanowire UV sensors on rigid and flexible substrates. Adv Funct Mater. 21:4464-4469


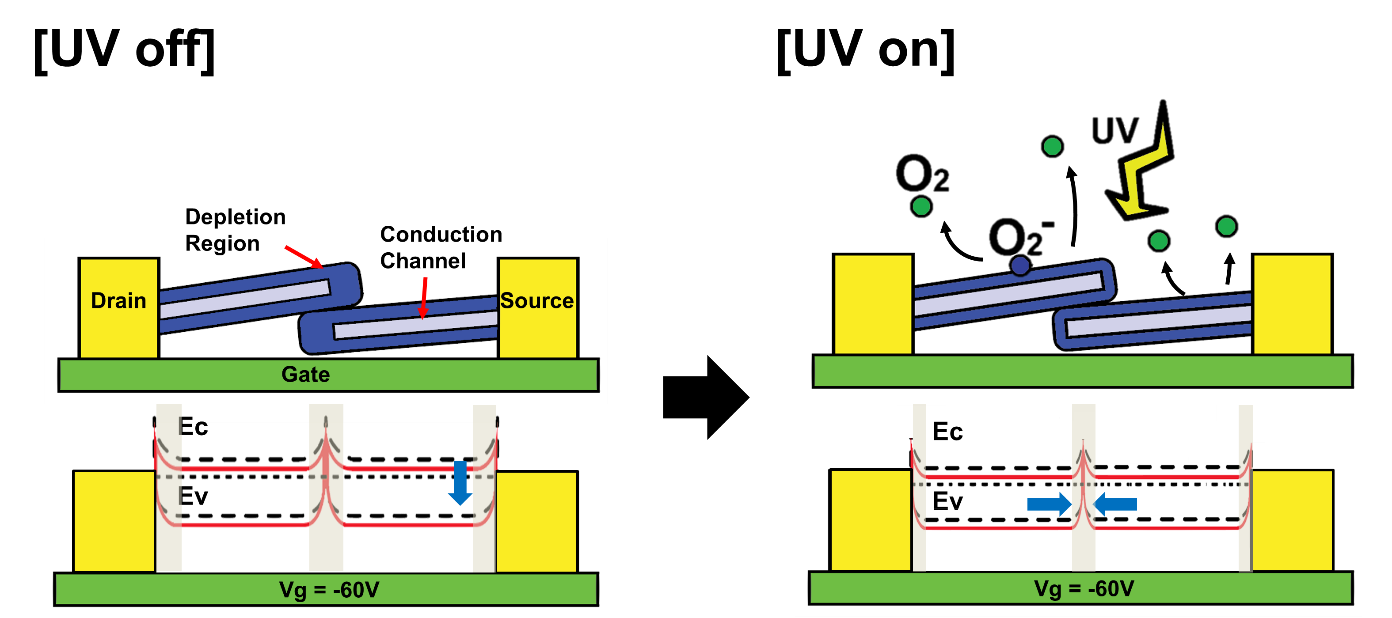


**Figure S9.** Schematic diagram depicting the carrier generation and transportation processes in the ZnO NW network channel before (left) and after (right) UV illumination. Band diagram of the devices under different gate bias conditions and UV illumination
